# Supplementary figures and images for: Suberoylanilide hydroxamic acid attenuates cognitive impairment in offspring caused by maternal surgery during mid-pregnancy
Source: PLoS One. 2024 Mar 29;19(3):e0295096. doi: 10.1371/journal.pone.0295096 (PMC10980197; doi:10.1371/journal.pone.0295096)

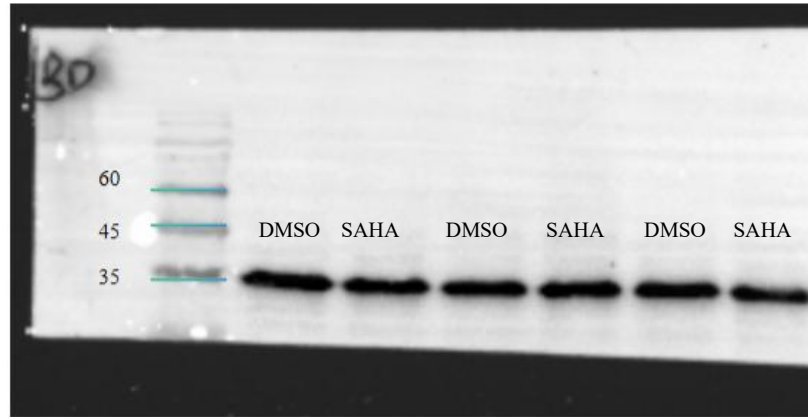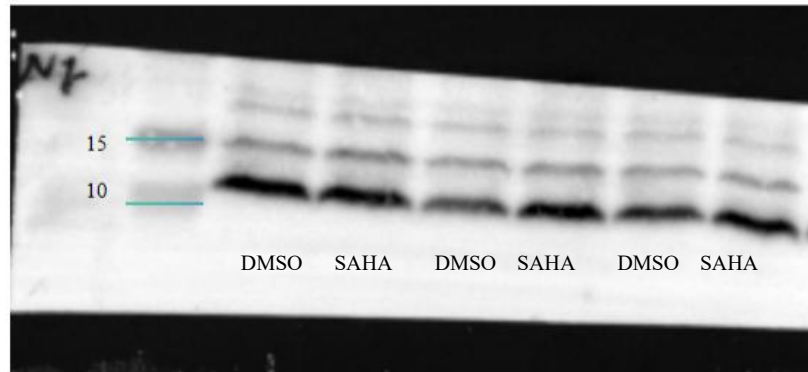

BDNF-15KD, GAPDH-35KD

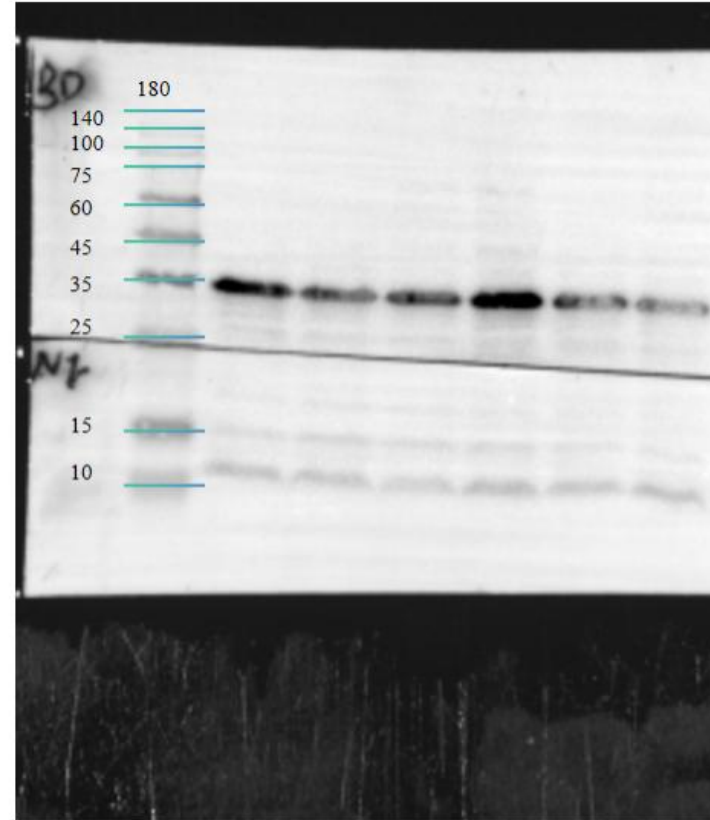

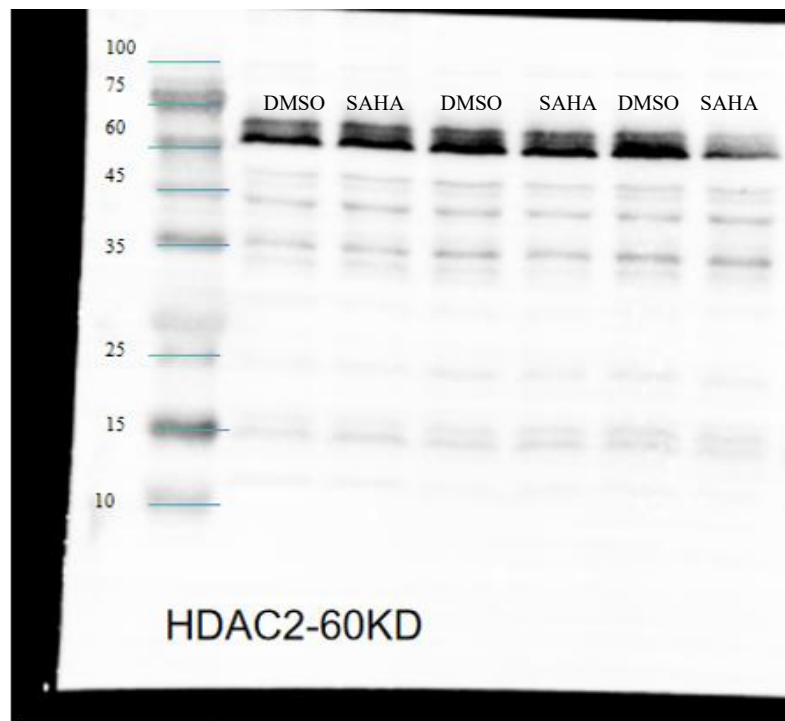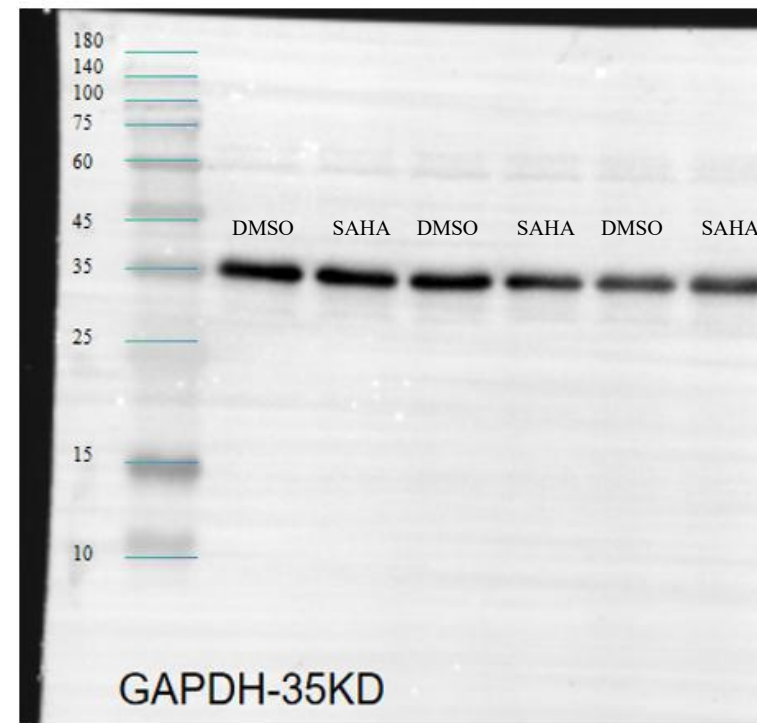

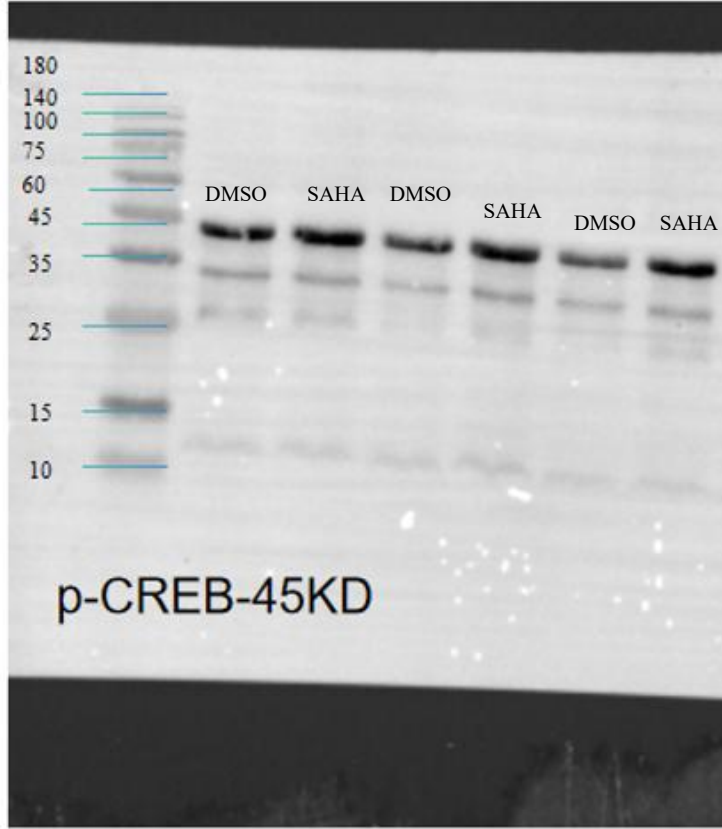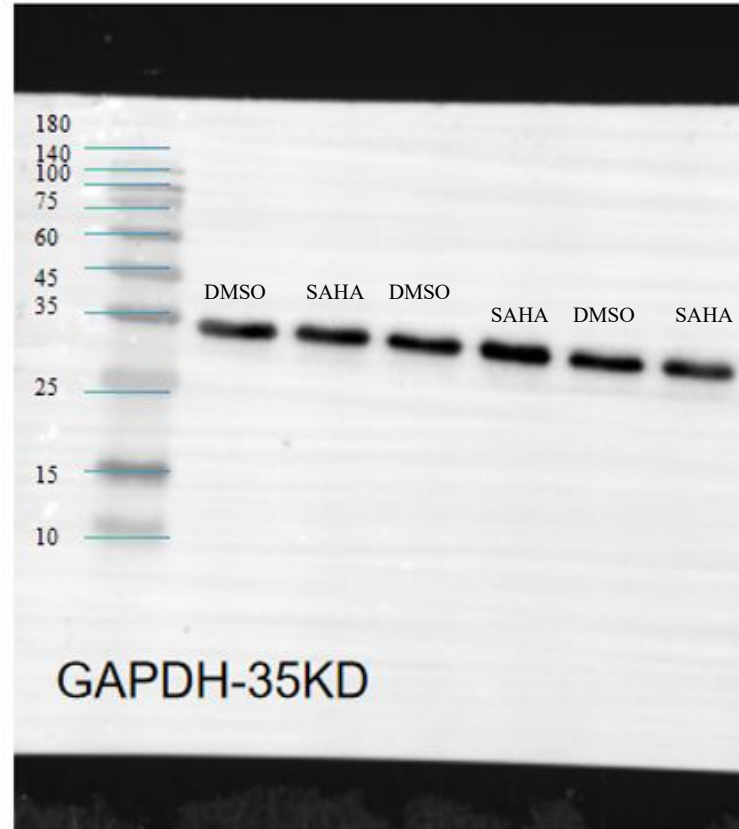

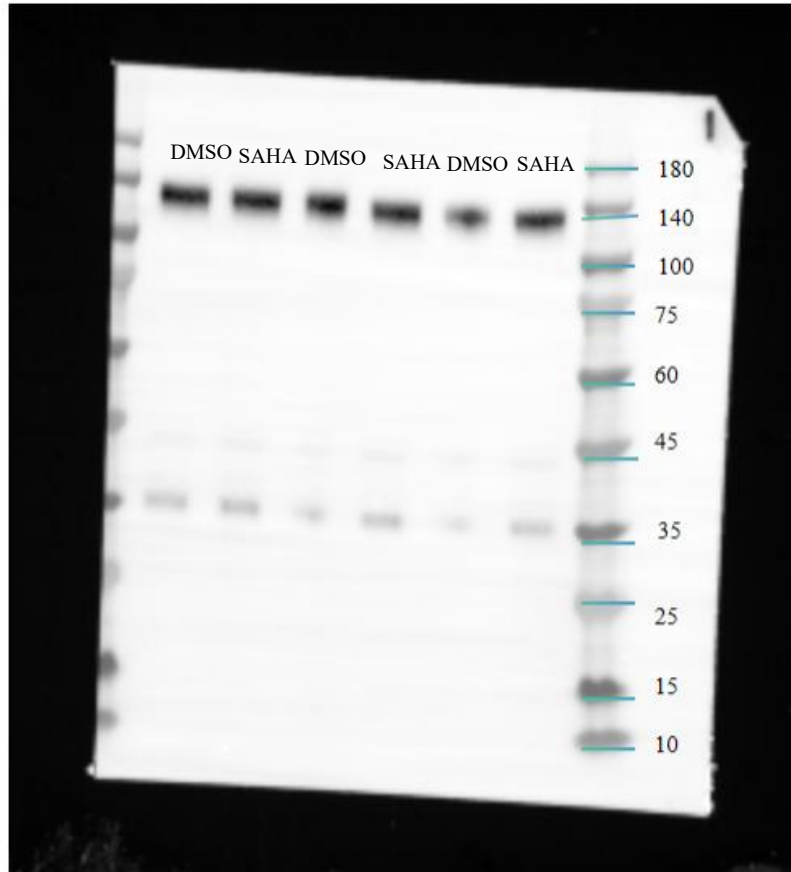

p-TrkB-145KD

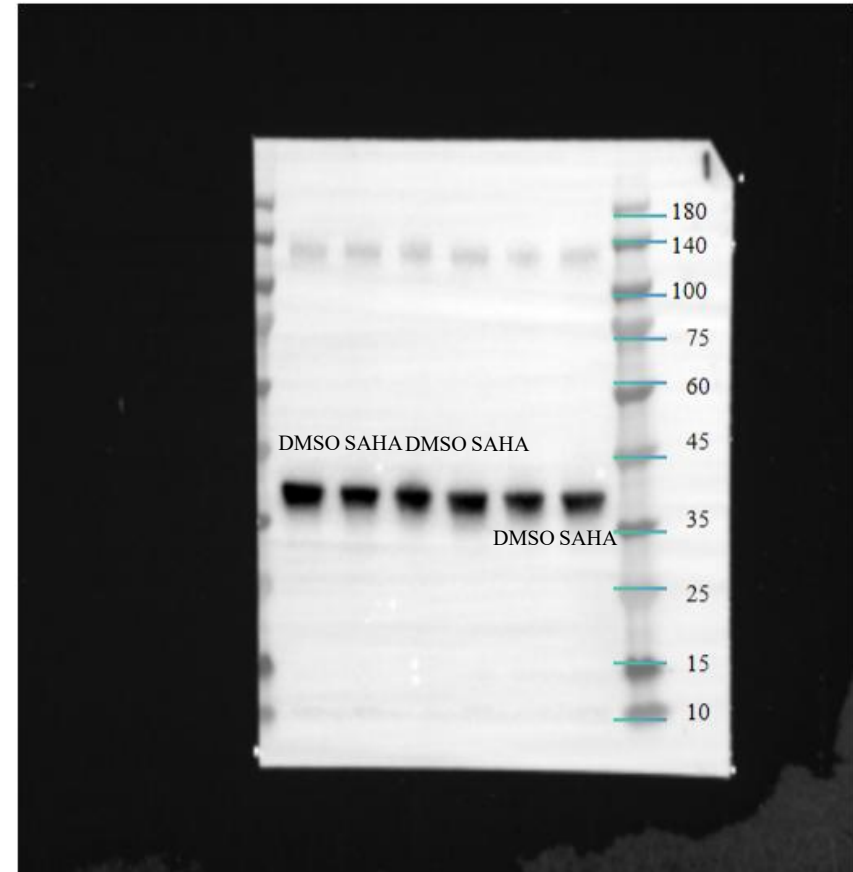

GAPDH-35KD

Supplement: S1 Raw images — (PDF) [file pone.0295096.s001.pdf]
