# Supplementary material for: Suberoylanilide hydroxamic acid attenuates cognitive impairment in offspring caused by maternal surgery during mid-pregnancy
Source: PLoS One. 2024 Mar 29;19(3):e0295096. doi: 10.1371/journal.pone.0295096 (PMC10980197; doi:10.1371/journal.pone.0295096)
Supplement: Attachment — Submitted filename: Response to Reviews..doc [file pone.0295096.s002.doc]

**Response to Review for PONE-D-23-37577**

**Dear Editor,**

We sincerely thank the editor and reviewers for their valuable feedback that we have used to improve the quality of our manuscript. We have now carefully addressed each of the comments/suggestions of the reviewers.

As below are our responses to reviewers’ comments/suggestions and our revisions to the manuscript (in blue font and italics). We believe addressing these comments/suggestions and revising the manuscript have significantly improved the quality of the paper. We hope that the manuscript in its present form could meet the standards of PLOS ONE.

**Editor**

The reviewers provide a variety of helpful comments regarding the presentation and discussion of the data. When preparing your revised version, please take them into account. Furthermore, for all bar graph presentations, I recommend that you also include the individual data points, a mode of presentation that is becoming the standard in the field. Furthermore, there is some language editing required. You may consider using an English language editing service.

*We appreciate for the editor's valuable suggestions. We have incorporated individual data points into all bar graph presentations, as suggested. Furthermore, we have addressed some language issues in our revised manuscript.*

**Reviewer #1**

The manuscript investigates the effect of SAHA on propofol-induced learning and memory deficits in offspring caused by maternal surgery during mid-pregnancy...... In general, the flow of the experiments is logical, and the findings are of considerable interest to the field. Based on the current results, the authors concluded that SAHA could be a potential and promising agent for treating learning and memory deficits in offspring caused by maternal nonobstetric surgery under propofol anesthesia. However, the current dataset should be enriched with a few supplemental details.

*Thank you very much for the positive comments on the manuscript.*

***Comment 1*:** In the fig4, the platform crossing times in the Propofol +DMSO group were less than that in the Control+DMSO group. There was no label for the statistical analysis of the two groups. Please further verify the original data and related statistical methods.

*The platform crossing time in the ‘Propofol +DMSO’ group was significantly less than that in the ‘Control+DMSO’ group. We have added the statistical label in Figure 4.*

***Comment 2*:** In the fig7, IF staining results revealed that HDAC2 is predominantly expressed in the hippocampal neuronal nucleus. Did the expression of HDAC2 in the hippocampus appear regional specificity? The HDAC2 should be detected by staining with neuronal biomarkers.

*The present study showed that HDAC2 has no regional specificity. It is extensively expressed in the neuronal nucleus of the hippocampus. We have conducted a new staining experiment to detect HDAC2 with the neuronal biomarker (NeuN). The result revealed that HDAC2 was co-expressed with NeuN-labeled neurons (see Figure 7A in the revised manuscript).*

**Reviewer #2**

This is a straightforward manuscript from Feng et al., entitled “Suberoylanilide Hydroxamic Acid Attenuates Cognitive Impairment in Offspring Caused by Maternal Surgery during Mid-pregnancy.” ... The data presented are convincing.

*Thanks a lot for the positive comments on the manuscript.*

However, a key piece of data that appears missing is the effect of propofol on some histone or non-histone acetylation marks linked to memory. Does propofol decrease acetylation? If so, SAHA would likely reverse such effects.

*Actually, we had presented the data regarding the effect of propofol on histone acetylation (see Figure 7 in the original submission). The result showed that propofol decreased acetylation of histone and SAHA reversed such effect (Figure 7C in the original submission and also in the revised manuscript).*

***Comment 1*:** The bar graphs in Figure 2 should have scattered points for the reader to better understand the spread of the data.

Revised, as suggested *(see Figure 2)*.

***Comment 2:*** Scattered points should also be added to Figures 3B, 3C, and 3D.

Revised, as suggested *(see Figure 3)*.

***Comment 3*:** Scattered points should be added to figures 4B, 4C, and 4D.

Revised, as suggested *(see Figure 4)*.

***Comment 4*:** It is not clear what significance is indicated in figure 4B for "propofol + SAHA". Is it a comparison between "propofol + DMSO" and "propofol + SAHA"? If so, it should be clearly indicated on the graph with brackets or other means.

*We have now marked the pair-wise comparisons in Figure 4B and revealed a significant difference between the "Propofol + DMSO" and "Propofol + SAHA" groups, as well as between the "Propofol+DMSO" and "Control+DMSO" (Figure 4B).*

***Comment 5*:** It would also be good to indicate whether the difference between "control + DMSO" and "propofol + DMSO" is significant. By eye, it looks as significant if not more than "propofol + DMSO" vs "propofol +SAHA."4B

*The difference between the "control + DMSO" and "propofol + DMSO" groups is now indicated, as suggested. (see Figure 4).*

***Comment 6*:** The same corrections as in Figure 4 are needed for Figure 5. More detailed graphical representations of the significance are needed for figures 5B and 5C.

*Figure 5 was revised, as suggested (see Figure 5).*

***Comment 7*:** Scattered points are needed for Figures 6B, 6C and 6D. Whenever there appears to be a slight difference, the stats should be graphically shown. For example, it could be "*" for a significance of p<0.05 or "ns" whenever p>0.05.

*Revised, as suggested. (see Figure 6).*

***Comment 8*:** Are figure 7C data normalized to DMSO control? If so the legend in the Y-axis should say **% DMSO control**. The same comment goes for figures 8C, 9C, 9D and 10B.

*The data in Figure 7C was normalized to the DMSO control and the legend in the Y-axis has been changed to“HDAC2 protein(% DMSO Control). Figures 8C, 9C, 9D, and 10B have been revised similarly.*

***Comment 9*:** Figure 7C graphically presents the stats well. It needs to be presented, however as bar graphs with scattered points. The same goes for figures 8C, 9C, 9D and 10B.

Revised, as suggested *(see Figure 7,8,9,10)*.

***Comment 10*:** The western blots used for quantification for figure 7C should be provided in the supplementary material. The same goes for Figures 8C, 9C and 9D.

*Provided in the original submission and re-submitted now, as suggested (see the supplementary material).*

***Comment 11*:** Do the authors have a hypothesis about why surgery exacerbates the effects of propofol? This should be addressed in the discussion. Could it be inflammation related?

*We have now included a paragraph in the revised "Discussion" section addressing why surgery exacerbates the effects of propofol (see below). We agree with the reviewer that this impairment could be associated with inflammation(lines 544-554).*

*“The combination of propofol anesthesia and surgery is a routine clinical condition. It is widely acknowledged that perioperative neurocognitive disorders (for example, learning and memory deficits) are associated with specific types of surgical procedures^[43]^. This kind of impairment could be due to inflammation. It has been reported that surgery activates a systematic cascade of inflammatory signaling molecules^[44]^and triggers local inflammatory responses. The postoperative elevation of inflammatory factors such as TNF-α, IL-6, IL-1β, and HMGB-1 could impair hippocampus-dependent learning and memory^[45]^, resulting in postoperative cognitive dysfunction (POCD^[46]^. Further investigations are needed for exploring the molecular and cellular mechanisms underlying the impact of maternal surgical procedures on offspring's cognitive function.”*

***Comment 12*:** Editing of the manuscript is needed. for example, the word "purchased" is erroneously separated as "pur" on line 105 and "chase" on line 106. Similarly, there is "laborator" on line 110 and "y" on line 111 to spell out "laboratory". There are many other instances of the same type of error. There are also many places where there is no space after periods, when a new sentence is starting.

*Thank you very much for pointing out the errors. We have corrected the errors you pointed out, and also made an effort to correct other spelling and grammar errors in the revised manuscript.*
